# Inferring gene regulatory networks from asynchronous microarray data with AIRnet

**DOI:** 10.1186/1471-2164-11-S2-S6

**Published:** 2010-11-02

**Authors:** David Oviatt, Mark Clement, Quinn Snell, Kenneth Sundberg, Chun Wan J Lai, Jared Allen, Randall Roper

**Affiliations:** 1 Department of Computer Science, Brigham Young University, Provo, UT, USA; 2 Department of Chemistry and Biochemistry, Brigham Young University, Provo, UT, USA; 3 Department of Biology, Indiana University-Purdue University Indianapolis, Indianapolis, IN, USA

## Abstract

**Background:**

Modern approaches to treating genetic disorders, cancers and even epidemics rely on a detailed understanding of the underlying gene signaling network. Previous work has used time series microarray data to infer gene signaling networks given a large number of accurate time series samples. Microarray data available for many biological experiments is limited to a small number of arrays with little or no time series guarantees. When several samples are averaged to examine differences in mean value between a diseased and normal state, information from individual samples that could indicate a gene relationship can be lost.

**Results:**

Asynchronous Inference of Regulatory Networks (AIRnet) provides gene signaling network inference using more practical assumptions about the microarray data. By learning correlation patterns for the changes in microarray values from all pairs of samples, accurate network reconstructions can be performed with data that is normally available in microarray experiments.

**Conclusions:**

By focussing on the changes between microarray samples, instead of absolute values, increased information can be gleaned from expression data.

## Background

Sequencing the human genome is one of the great accomplishments in recent history. The knowledge gained through sequencing the human genome is vast and holds great implications for medical practice [1]. No single gene, however, decides how an organism grows and matures. Genes form regulatory networks where many genes interact to produce an observable phenotype [2,3]. An understanding of gene regulatory networks is the key that will open the door to major breakthroughs in fields as diverse as agriculture [4-6] and medicine [7-11].

Many factors can influence each gene’s expression at any moment. One or more proteins produced by other genes within the regulatory network can promote or inhibit the expression of a particular gene. An understanding of how genes interact with each other is essential to developing new drugs and treatments. In many studies where gene expression data is used, tens of samples from a diseased organism will be compared with tens of samples from normal individuals. Average values from these two pools may not show statistically significant fold changes because the expression value for a gene may naturally vary significantly between individual samples at different time points. It can be difficult to infer signaling information based on these average values.

As an illustration of this problem, imagine a car race. Two of the drivers have a wireless headset that allows them to communicate. Although they may never be in the same absolute position at the same time, their velocity and acceleration could be correlated as they signal each other through their headsets. If you averaged the position of all cars throughout the race, these two cars may not appear to be more correlated in their position to each other than any other cars in the race. If you examine their velocity and acceleration, however, these two cars would appear to be much more correlated than other cars who may have more similar positions. The same effect can be observed in microarray data. The samples collected during an experiment may not show a correlation in their average values, but the changes (velocity and acceleration analogs) may be correlated. This correlation in changes can be an indicator that the genes are signaling each other, or that they are both being modulated by an external effect. This correlation can generate new hypotheses for connections that can be validated through biological experiments.

Asynchronous Inference of Regulatory Networks (AIRnet) unravels the complexity of regulatory networks using unsynchronized microarray data that is generally available to researchers to create a network based on the correlation of gene expression changes between microarray experiments. Expression values for all pairs of samples in a data set are compared to determine the correlation in changes between all sets of genes in the sample. Edges between genes whose changes appear to be correlated in the largest percentage of microarray pairs are inserted into the inferred gene regulatory network. AIRnet can then compare the network for the diseased and normal data sets to determine which pathways are being disrupted by the condition.

AIRnet has produced promising results when inferring realistic *in-silico* regulatory networks. In experiments with real mouse arrays, AIRnet has also produced a significant number of validated connections. Research is currently underway to validate predicted connections through in-vivo techniques.

### Related work

Many strategies have been formulated to deduce gene regulatory networks from microarray data. In a paper written by Wang *et al.*[12], a strategy is proposed that uses multiple microarray samples from different experiments to find a gene regulatory network. Each of these data sets represents a unique experiment. Each experiment is assumed to represent a unique perturbation to the gene regulatory network. Gene regulatory networks are also assumed to be sparse. Differential equations are used to derive a general solution that is the best representation of the invariant parts of the different microarray data sets. Their results show that they are successful in reconstructing small networks. Our algorithm, unlike Wang *et al.,* does not utilize differential equations to form a model of the regulatory network, but employs a much simpler method that can be extended to whole genome studies. While differential equation based inference techniques are limited to tens of genes, AIRnet has been shown to be effective with 44,000 gene probes. Another popular strategy developed by Liao *et al.*[13], called network component analysis, makes assumptions about power law relationships between genes and the factors that influence their expression. They explain that microarray data is frequently given as a log ratio, thus being pseudo-linear. Then, based on these premises, a regulatory network can be written as *E* = *A* * *P*. Where *E* is the microarray data, *A* represents prior information about the network, and *P* represents samples of regulatory signals. When there is no noise associated with this relationship, there is a unique analytic solution that can be found. In real applications, there is noise, and through the use of simulated and real data they are able to reconstruct gene relationships with acceptable accuracy. Their results depend largely on the amount of noise present. A shortcoming of this approach is that prior information about a network needs to be known and expressed in matrix form. Another problem is that there are very stringent constraints on the characteristics of matrix *A. A* has to have full column and row rank, and if any connections are removed, *A* still has to have full column and row rank. These restrictions make this method cumbersome to use and limits the datasets that it can be applied to. AIRnet’s algorithm can be applied to any set of microarray data and will extract as much of the signal that is present in the data.

A third method for discovering gene regulatory networks has been implemented by Nathan Barker [14] in iBioSim. His method first divides the microarray expression data into three categories; high, medium and low. Each gene is assigned one of these values based on its relative expression level when compared to each of the other genes’ expression levels. This categorization of genes assigns an approximately equal number of genes to each of the three categories. Barker’s algorithm then uses these categories to build an influence vector that shows the degree of influence each gene at one time point has on every other gene in the next time point. Barker’s algorithm assumes it has time series data when comparisons between samples are made. This assumption allows the algorithm to decide which gene is promoting or inhibiting another, rather than just find that there is a promoting or inhibiting relationship between two genes.

AIRnet takes a new approach to microarray analysis that avoids many of the pitfalls of existing approaches. The focus of the algorithm is on correlation between the changes of different genes instead of instantaneous probe values. This approach can be extended to full genome microarray studies, where differential equation based approaches are limited to small numbers of genes. AIRnet does not require prior information about gene interactions and through examining correlation in the changes in gene expression, it does not require time series data. Methods that require time series data are not practical with real microarray data. The simple fact that several samples are taken sequentially does not guarantee that they are sequential as far as the biological model is concerned. The time taken for genes to interact with one another is too small for us to accurately measure, and certainly too small to be able to generate a microarray sample at each time step, which would be necessary to fulfill time series assumptions.

Other methods of microarray analysis are based on statistical significance tests. Gene Set Enrichment Analysis, proposed by Subramanian *et al.*[15], and Significance Analysis of Microarray Gene Sets, proposed by Dinu *et al.*[16], are two methods that apply statistical tests to previously selected groups of genes, such as genes in the same signaling pathway. These methods attempt to identify whether the gene sets are associated with a particular phenotype. Similarly, AIRnet can highlight a subset of genes within the entire network, either treating the subset as the entire network, or showing only genes, which are highly correlated with one or more genes in the specified subset. AIRnet will also compare networks to identify phenotype-specific gene correlations.

## Results and discussion

Results were gathered from *in-silico* regulatory network data originally created for the DREAM3 competition [17]. This data was generated from a TRUE network, so inferred networks can be compared on an edge by edge basis to the original network. Although this kind of comparison is easy to perform, it lacks some biological reality. The second set of results was generated by examining real mouse microarray data. Edges in the network inferred by AIRnet were validated through public pathway databases. This approach is limited because a legitimate gene relationship inferred by AIRnet may not have been studied enough to make its way into a publication. But these results show that the accuracy of the algorithm is not limited to simulated data.

### In-silico validation

**Figure 1:**
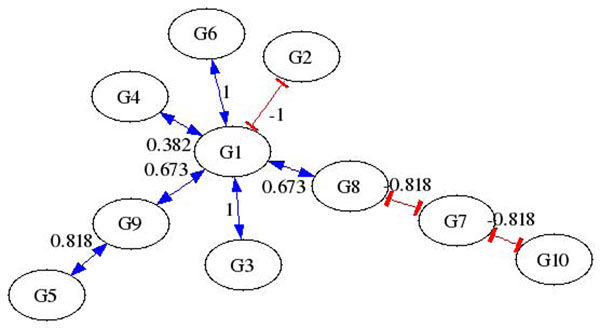
Sample AIRnet Network inferred using 40% threshold

**Figure 2:**
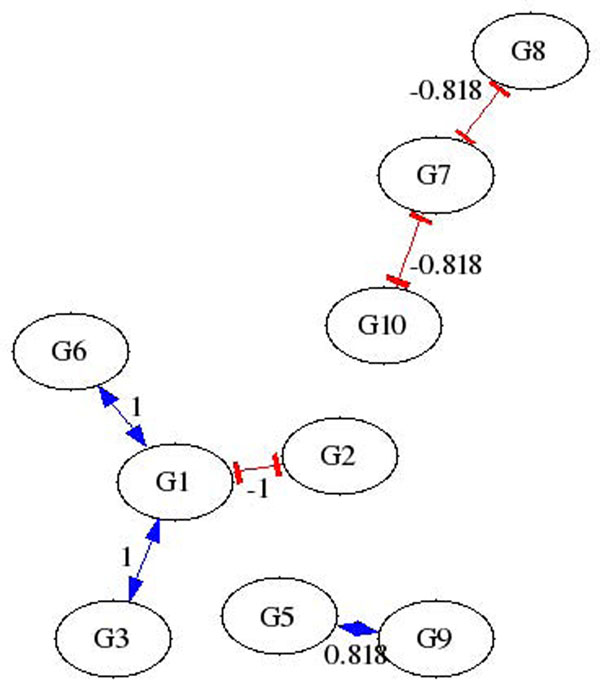
Sample AIRnet Network inferred using 80% threshold

**Figure 3:**
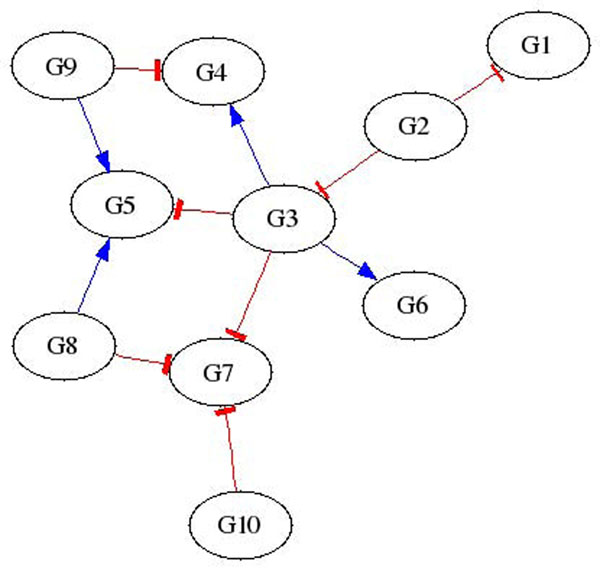
True in-silico network

Three types of data are used to test AIRnet’s accuracy for each of the *in-silico* regulatory networks. The data types are labeled as heterozygous knock-down, null-mutant, and trajectory. The heterozygous knock-down and null-mutant data sets each contain data for the steady states of the wild-type as well as knock-down or knock-out data for each gene. Trajectory data sets are comprised of time series data, with 21 time points, for each network recovering from external perturbations. Each network is subjected to, and has data for, a number of perturbations equal to 46% of the number of genes within the network. Figures 1 and 2 depict regulatory networks inferred by AIRnet using data produced by one of the networks generated for DREAM3. The *in-silico* network is shown in Figure 3. Figures 1 and 2 show that higher threshold values produce more selective networks by excluding connections for which the correlation between the two genes is not great enough. Visual verification, however, is not always the best method for measuring the accuracy of an inferred regulatory network, especially if the network is large.

Scoring metrics from the DREAM3 competition are used to verify the statistical significance of AIRnet’s reconstructed regulatory networks. The DREAM3 metrics calculate the AUROC and AUPR values and compare the resulting values with the AUROC and AUPR of 100,000 randomly generated networks to compute the probability of randomly creating a network with equal or greater AUROC and AUPR values, producing a p-value for both the AUROC and AUPR. The AUROC p-values are combined by averaging the scores for same-sized networks. The same is done for the AUPR p-values. The averaged AUROC and AUPR p-values are subsequently combined as a log-transformed average, –*log10*(*AU ROC_p_* * *AUPR_p_*)/2. Each log-transformed average provides a single value, which summarizes AIRnet’s accuracy for five individual, same-sized networks.

Because the graphs AIRnet produces are signed and undirected, the standards, against which AIRnet is being measured, were modified to be undirected as well.

The score, along with the AURR and AUROC *p*-values, are displayed in Table 1. The first row in each section of Table 1, the empty network, report the values obtained from a network with zero edges, or a network which assumes genes do not interact in any way with each other. The empty network is not produced by AIRnet, but is included as a baseline for comparing AIRnet’s accuracy using the supplied data types. Other rows correspond to the type of data used to infer the networks, as specified by the first column.

As seen in Table 1, the null-mutant data produces significantly better results than either of the other two data types. The networks AIRnet infers using null-mutant data appear to be only marginally better when inferring small networks, however, as the network size grows, the null-mutant produced networks’ accuracy grows at a much faster rate than the accuracy for networks produced by either the heterozygous data, or the trajectory (time series) data.

It is interesting to note, using the trajectories data to infer networks gave the lowest scores of all the data types, in one case, scoring even lower than the empty network even though the data was produced by a simulator with accurate time series outputs (Table 1).

Comparing the values in Table 1 with the 300 submissions to the DREAM3 competition ranks AIRnet in the top 5 performers. This comparison ignores directionality, which would probably lower the AIRnet ranking. The results are promising and more importantly, are obtained using microarray assumptions that can be met by most biological experiments.

**Table 1:** AIRnet results for DREAM3 competition

*Values for 10-gene networks.*			
	score	AUPR *p*-value average	AUROC *p*-value average

empty network	1.1816e+00	8.5675e-03	5.0578e-01
trajectories	1.6298e+00	2.1759e-03	2.5279e-01
heterozygous	2.2401e+00	3.6441e-04	9.0845e-02
null-mutant	2.8198e+00	4.1550e-05	5.5198e-02

*Values for 50-gene networks.*			

	score	AUPR *p*-value average	AUROC *p*-value average

empty network	2.4438e+00	2.6065e-05	4.9687e-01
trajectories	2.6700e+00	6.1865e-06	7.3901e-01
heterozygous	2.6207e+00	7.7215e-06	7.4297e-01
null-mutant	1.4152e+01	5.2984e-26	9.3634e-04

*Values for 100-gene networks.*			

	score	AUPR *p*-value average	AUROC *p*-value average

empty network	5.2312e+00	6.8572e-11	5.0297e-01
trajectories	3.8264e+00	4.7395e-08	4.6923e-01
heterozygous	5.2881e+00	6.3523e-11	4.1762e-01
null-mutant	3.7911e+01	1.0263e-71	1.4694e-05

AIRnet results for 3 network sizes from the DREAM3 competition. The empty network row shows values for graphs with 0 edges and provide a baseline to interpret the scores for other networks. The empty network scores were generated by submitting an empty file with no predictions to the DREAM score evaluator. The other rows correspond to the type of data used to infer the networks. In the score column, larger values are better. In the other two columns, smaller values are better. Scores reported are produced using an 80% threshold parameter for AIRnet.

### Real microarray data

Microarray data was examined from an experiment with 11 euploid and 13 trisomic Ts1Cje mice to show the utility of AIRnet with real data. The euploid arrays were examined separately from the trisomic arrays and the resulting regulatory networks were then compared. One important feature of the AIRnet approach is the increased number comparisons that are possible. With 11 euploid arrays, there are 110 different pairs that can be examined for changes. If the data were treated as a time series, then only 10 comparisons could be made. If mean values were used, then there would be only one comparison between the euploid and trisomic groups. With 13 trisomic arrays, there are 156 pairs of arrays.

The AIRnet analysis found 566 genes with significantly correlated changes in expression between arrays. Each edge in this network was then submitted to PathGen [18] to determine which inferred connections have been verified through wet-lab experiments. The PathGen database was created using data from KEGG PATHWAY, PubMed abstracts, DIP (Database of Interacting Proteins), HPRD, Reactome, MIPS, BIND, IntAct and MINT. There are currently, over two million interactions included in the PathGen database. PathGen returned the number of intermediate nodes in the pathway found in the database corresponding to a direct connection inferred by AIRnet. The results in Table 2 show that nearly 70% of the interactions inferred by AIRnet exist with some number of intermediate genes in a public regulatory network database. It is difficult for most inference algorithms to identify all of the hops between two genes that are highly correlated. For this reason, we count an inferred connection as validated if there is multi-hop connection in a pathway database.

**Table 2:** AIRnet results on experimental data

Intermediate Genes	Connections	Percentage
1	148	0.41%
2	3110	8.71%
3	12253	34.34%
4	8065	22.60%
5	1349	3.78%
**Validated AIRnet Connections**	**24926**	**69.87%**
**Validated Random Network Connections**	**17841**	**27.90%**

AIRnet inferred connections found in public databases. The table shows the number of connections in the AIRnet inferred network that have the given number of intermediate connections. A Connection count of one indicates that the genes are directly connected in the graph. A count of two indicates that the genes have one intermediate gene between them in the pathway database. The percentage shows the percentage of connections in the inferred network that have this Connection count. AIRnet connections were validated almost 70% of the time, where less than 30% of the connections in random networks were validated.

An inference rate of 70% is extremely good when compared to other inference algorithms. In order to quantify this quality an experiment was performed with 100 random networks of 566 genes. The same number of connections were made in these random networks as exist in the AIRnet results. These edges were then submitted to PathGen to determine how AIRnet results would compare to random networks. Less than 30% of the connections in random networks were found in pathway databases, showing that AIRnet infers connections that are significantly more likely to be real than random networks. The PathGen database is biased towards more studied regulatory pathways, so actual accuracy of AIRnet is probably higher than the 70% reported.

**Figure 4:**
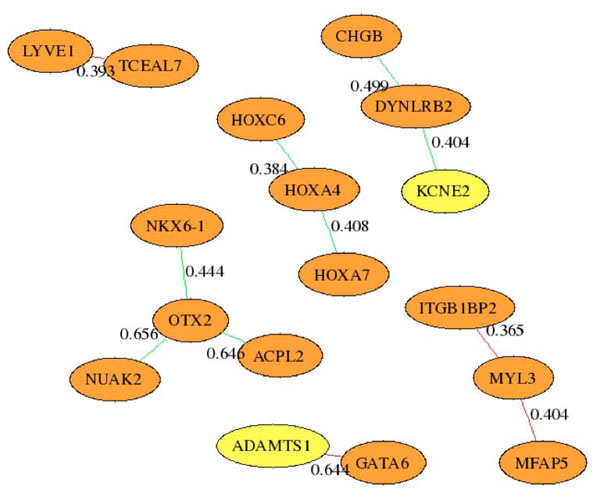
Differences between Euploid and Trisomic regulatory networks inferred by AIRnet. Numbers on edges are the absolute value of the difference between the edge weight in Euploid and Trisomic samples. Red edges were found only in Euploid and Green edges were found only in Trisomic samples. Genes with three copies in the trisomic Ts65DN mice are shown in yellow.

Another benefit of inferring a regulatory network based on changes in expression values between microarrays is the ability to compare the network generated by diseased and normal arrays. In this experiment we compared the network generated by the 11 euploid microarrays with the network generated by the 13 trisomic arrays. The results are plotted in Figure 4. The edge between ADAMTS1 and GATA6 is found only in the Euploid inferred network. This may indicate that having three copies of the trisomic gene ADAMTS1 may disrupt this connection to GATA6. Further research is being performed to validate this kind of hypothesis.

## Conclusions

AIRnet infers regulatory networks from microarray data with practical assumptions. The microarray data does not have to have time-series characteristics and no constraints are placed on the structure of the matrices. Networks inferred by AIRnet are comparable in accuracy to the best algorithms participating in the DREAM3 competition even though many of these algorithms were more restrictive on the kind of data they could use. Edges predicted by AIRnet also compare favorably with experimentally validated regulatory networks found public databases [18]. Perhaps the most important aspect of this approach is scale. AIRnet can perform predictions on microarrays with 44,000 gene probes in less than 24 hours, making it practical for most analysis needs. This new approach of looking at correlation in changes, rather than comparisons of mean values can provide the understanding of gene regulatory networks necessary for the impending major breakthroughs in agriculture and medicine.

In the analogy where race car drivers were signaling to each other to change velocity, an examination of instantaneous positions would not be able to detect this signaling. Only by looking at velocity could the correlation be detected. AIRnet examined the analogous velocity of changes in microarray data to detect correlations that can not be inferred by methods that focus on absolute values. AIRnet can be downloaded from http://dna.cs.byu.edu/airnet/

## Methods

AIRnet infers a gene regulatory network by analyzing how genes change between microarray samples instead of focussing on their absolute values. Non-time series data from different samples are used to find correlated patterns in the way genes change. The samples collected during an experiment may not show a correlations in their average values, but the changes may be correlated. This correlation in changes can be an indicator that the genes are signaling each other, or that they are both being modulated by an external effect. The correlation of gene expression changes is used to create influence vectors, which highlight the genes with the highest probability of being correlated. Ultimately, AIRnet is designed to compare two networks of different genotypes, in order to draw out differences that will assist researchers with the development of treatments.

The first step in analyzing microarray data is discretizing the data (Table 3). While discretizing, the data for a single gene across all the samples is considered a single dataset. Each dataset is clustered using *k*-means clustering. After all the data sets have been discretized, the data for each gene in every sample is classified as a number between 0 and *k* – 1. The new value represents the relative level of activation for a particular gene, as compared between samples. The example in Table 3 shows data being discretized into two values (0 and 1), but users can select a larger number of discrete values for the discretization step. After discretizing the data, AIRnet performs a pairwise comparison of the change in activation state between samples for all genes.

Comparing genes *x* and *y*, AIRnet calculates an influence vector, *v_xy_*, representing how correlated *x* and *y* appear to be (*equations 1 and 2*). An influence value is generated for each pair of microarrays and the vector contains the set of these values. Negative values of *v_xy_* indicate a inhibiting relation (negatively correlated) between *x* and *y*, while a positive value of *v_xy_* indicates a promoting relation (positively correlated). Several other similarity metrics were examined, but this method provided the highest accuracy. Commonly used metrics such as the Pearson correlation coefficient do not apply well when looking for correlation of changes instead of correlation of absolute values.

**Table 3:** Data discretization

*Pre-discretized data for an* in-silico *regulatory network consisting of 5 genes*									
	wt	G1(-/-)	G2(-/-)	G3(-/-)	G4(-/-)	G5(-/-)	G6(-/-)	G7(-/-)	G8(-/-)

G1	0.105	0.034	0.927	0.088	0.015	0.049	0.102	0.105	0.018
G2	0.877	0.804	0.000	0.864	0.870	0.981	0.837	0.873	0.797
G3	0.054	0.000	0.838	0.000	0.103	0.000	0.069	0.000	0.085
G4	0.386	0.310	0.611	0.243	0.083	0.432	0.440	0.394	0.364
G5	0.801	0.808	0.748	0.903	0.793	0.000	0.880	0.741	0.686

*Post-discretized data for the same* in-silico *regulatory network consisting of 5 genes, k* = 2.									

	wt	G1(-/-)	G2(-/-)	G3(-/-)	G4(-/-)	G5(-/-)	G6(-/-)	G7(-/-)	G8(-/-)

G1	0	0	1	0	0	0	0	0	0
G2	1	1	0	1	1	1	1	1	1
G3	0	0	1	0	0	0	0	0	0
G4	1	0	1	0	0	1	1	1	1
G5	1	1	1	1	1	0	1	1	1

Discretization of data by k-means clustering for an *in-silico* network consisting of 5 genes [17] - genes are divided by row, samples are divided by column.

 (1)

 (2)

The Dialogue for Reverse Engineering Assessments and Methods (DREAM) organization creates benchmark data and metrics for evaluating the accuracy of gene regulatory network inference algorithms [17]. Table 4 shows example data for generating influence vectors. The columns represent different microarray samples for the DREAM knockout experiment. In the first section of Table 4, G1 changes from a 0 to a 1 between sample G1(—/—) and G2(—/—). The same change is observed for G6. This would represent one vote for positive correlation for G1 and G6.

**Table 4:** Activation state change examples

*equal, non-zero activation state changes - v_xy_ incremented*									
	wt	G1(-/-)	G2(-/-)	G3(-/-)	G4(-/-)	G5(-/-)	G6(-/-)	G7(-/-)	G8(-/-)

G1	0			0	0	0	0	0	0
G2	1	1	0	1	1	1	1	1	1
G3	0	0	1	0	0	0	0	0	0
G4	1	0	1	0	0	1	1	1	1
G5	1	1	1	1	1	0	1	1	1
G6	0			0	0	0	0	0	0

*equal magnitude, opposing-signed activation state changes - v_xy_ decremented*									

	wt	G1(-/-)	G2(-/-)	G3(-/-)	G4(-/-)	G5(-/-)	G6(-/-)	G7(-/-)	G8(-/-)

G1	0	0			0	0	0	0	0
G2	1	1			1	1	1	1	1
G3	0	0	1	0	0	0	0	0	0
G4	1	0	1	0	0	1	1	1	1
G5	1	1	1	1	1	0	1	1	1
G6	0	0	1	0	0	0	0	0	0

*activation state changes equal to zero - q_xy_ incremented*									

	wt	G1(-/-)	G2(-/-)	G3(-/-)	G4(-/-)	G5(-/-)	G6(-/-)	G7(-/-)	G8(-/-)

G1	0	0	1	0	0	0	0	0	0
G2	1	1	0	1	1	1	1	1	1
G3	0	0	1	0	0	0	0		
G4	1	0	1	0	0	1	1		
G5	1	1	1	1	1	0	1	1	1
G6	0	0	1	0	0	0	0	0	0

Activation State Change Examples - genes are divided by row, samples are divided by column

The second section of Table 4 illustrates an example of support for negative correlation. G1 changes from a 1 to a 0 between sample G2(—/—) and G3(—/—). G2 changes from a 0 to a 1 between sample G2(—/—) and G3(—/—) providing evidence for negative correlation.

In the third section of Table 4, G3 and G4 stay the same between sample G7(—/—) and G8(—/—). This would represent one vote for positive correlation for G3 and G4 since neither gene changed, even though their values are different. Change values for all pairs of samples are summed to generate the influence vector for a pair of genes.

Given a practical microarray experiment with 20 samples, time series based algorithms would only be able to perform 19 comparisons between values, even if the experiments were performed with a perfect time period between samples. Due to the asynchronous nature of AIRnet inference, all pairs of samples can be used, resulting in 190 comparisons. Even with 10 samples, 45 comparisons can be performed in to determine which gene expression values are correlated in their changes.

Following the calculation of *v_xy_* for all values of *x* and *y*, AIRnet reconstructs the regulatory network by including edges that have the highest number of pairs of experiments where the expression changes are correlated. In our example with 10 experiments, there are 45 pairs of samples. If Gene A and Gene B both increased in 20 of these pairs and both decreased in 20 of the pairs and were uncorrelated in 5 of these pairs, then the degree of correlation would be (20 + 20)/45 = 0.88. The edges between genes with the highest degree of correlation are included in the reconstructed network.

AIRnet produces a graph, *G* representation of the regulatory network, where each node, *x*, represents a single gene, and each edge, ({*x*, *y*}, *v*) represents an interaction between *x* and *y*. The sign of *v* shows the interaction between *x* and *y* as either promoting (positively correlated) or inhibiting (negatively correlated), while |*v*| shows the probability of *x* interacting with *y*. To form the graph, AIRnet adds the edges ({*x*, *y*},*w_xy_*), where *w_xy_* = 1 – |*v_xy_*|, for all values of *x* and *y*. To prune edges out of the graph, Kruskal’s Algorithm is used to find the minimum cost spanning tree of the graph *G*, with the addition of stopping the production of the minimum cost spanning tree when the value of |*w_xy_*| for the next edge to be added falls below a user-defined threshold. The *w_xy_* values are then exchanged with their corresponding *vxy* values. This algorithm creates a graph with edges between genes that are most highly correlated in their changes. This graph is similar to the mutial information graph produced by MINET [19].

## Authors contributions

DO implemented the algorithm, wrote the documentation and created the source distribution. MC participated in the design of the algorithm and supervised the validation. QS participated in the design of the algorithm and validation. KS designed an initial version of the algorithm and helped in creating the companion program used for validation. CWL performed the validation with pathgen. JA performed trisomic mice experiments and compared results with other applications. RR provided vision for algorithm development and specification for program results. All authors read and approved the final manuscript.

## Competing interests

The authors declare that they have no competing interests.

## Acknowledgements

Brigham Young University funded this research through a Mentoring Environment Grant. Purdue University Indianna University Indianapolis also provided travel funding for the collaboration. Publication of this supplement was made possible with support from the International Society of Intelligent Biological Medicine (ISIBM).

This article has been published as part of *BMC Genomics* Volume 11 Supplement 2, 2010: Proceedings of the 2009 International Conference on Bioinformatics & Computational Biology (BioComp 2009). The full contents of the supplement are available online at http://www.biomedcentral.com/1471-2164/11?issue=S2.
